# Nurses' knowledge and attitudes towards medical device-related pressure injuries in intensive care and surgical units: Cross-sectional study

**DOI:** 10.1371/journal.pone.0356550

**Published:** 2026-08-19

**Authors:** Berna Dizer, Nilufer Ozgurbuz, Arzu Bahar, Murat Runa

**Affiliations:** 1 Vocational School of Health Services, Operation Room Services Department, Izmir Tınaztepe University, Izmir, Turkey; 2 Independent Researcher, Izmir, Turkey; 3 Faculty of Health Sciences, Nursing Department/Fundamentals of Nursing, Çankırı Karatekin University, Çankırı, Turkey; 4 Department of Internal Medicine, Intensive Care Unit, Harran University Hospital, Şanlıurfa, Turkey; King Khalid University, SAUDI ARABIA

## Abstract

**Objective:**

Medical device-related pressure injuries (MDRPIs) are a common complication in healthcare, leading to increased healthcare costs, prolonged hospital stays, and decreased quality of life. This study aimed to assess the knowledge and attitudes of nurses in these units regarding MDRPIs to identify gaps and inform preventive nursing practices.

**Methods:**

This cross-sectional study was conducted between 15 November 2022 and 30 December 2022 in the surgical and intensive care units of a state hospital. Data were collected from 150 nurses using a structured questionnaire assessing MDRPI-related knowledge and attitudes (MDRPIKAQ). Statistical analyses were performed to evaluate nurses’ knowledge and attitudes toward MDRPIs.

**Results:**

The mean knowledge score was 24.33 ± 5.20, and the mean attitude score was 16.64 ± 3.48. According to the MDRPIKAQ cutoff score (≥45/80, indicating adequate knowledge and positive attitudes), approximately one-third of nurses scored at or above this threshold. Item-based analysis indicated persistent misconceptions regarding MDRPI prevention and healing.

**Conclusion:**

Nurses demonstrated moderate knowledge and generally positive attitudes toward MDRPI prevention, although important misconceptions remain. Knowledge and attitude scores varied across selected characteristics, but no statistically significant association was observed between the total scores. These findings highlight the potential value of structured, MDRPI-focused education within nursing education and institutional training programs. However, because this was a single-center cross-sectional study, the generalizability of the findings is limited.

## Introduction

Pressure injuries (PIs) are recognized as significant care-related complications in healthcare institutions and are associated with increased healthcare costs, prolonged hospital stays, higher morbidity and mortality, and reduced quality of life their prevalence remains substantial among hospitalized patients, particularly in high-risk settings such as intensive care units (ICUs) [1–3]. PIs are also frequently observed in surgical patients during the perioperative period, where anesthesia, immobility, and prolonged operative time increase risk [4,5]. The Association of perioperative Registered Nurses defines surgical site pressure injury as a pressure injury that occurs within the first 48–72 hours after surgery [6]. In patients undergoing surgery, PIs may develop very early; for example, one study reported that Stage I PIs occurred immediately after surgical intervention in 9.8% [7]. Key perioperative factors associated with pressure injury development include anesthesia- and surgery-related characteristics, patient positioning, hemodynamic instability, and the use of perioperative devices and support surfaces [5,8].

Similarly, patients admitted to ICUs are at risk of PIs due to decreased sensory perception and mobility, continuous exposure of the skin to moisture (e.g., due to urinary incontinence) and the use of various medical devices [9–10]. Medical devices used for diagnosis and treatment are frequently associated with PIs in high-risk patient groups [11–14]. Reported incidence rates of MDRPIs vary across clinical settings, ranging from approximately 5% to over 30% in acute and intensive care environments [14,15]. A recent meta-analysis including over 117,000 patients reported a pooled MDRPI incidence of 19.3%, with higher rates observed in ICUs [2]. Similarly, a 2023 international meta-analysis of adult ICU patients involving 10,084 individuals from 11 countries estimated pooled incidence and prevalence rates of 14.7% and 19.0%, respectively, and identified mechanical ventilation as a major risk factor [16]. These findings highlight the substantial clinical burden of MDRPIs, particularly in high-acuity settings. Moreover, the presence of medical devices has consistently been identified as an independent risk factor for the development of PIs in hospitalized patients [2,16].

The use of medical devices made of rigid materials such as splints, anti-embolic stockings, foley catheters, nasogastric tubes, and saturation probes, as well as incorrect device selection and neglect of interventions to prevent cutting forces caused by the device, contribute to the development of MDRPIs [11,17,18]. Intensive care patients are at increased risk for MDRPIs due to prolonged immobility, sedation, and exposure to life-sustaining medical devices. A recent international meta-analysis reported pooled incidence and prevalence rates of 14.7% and 19.0% in adult ICU populations, identifying mechanical ventilation as a major independent risk factor (OR = 9.67) [16]. Similarly, surgical patients are considered high-risk due to prolonged operative time, anesthesia-related factors, and perioperative hemodynamic instability [5,8]. Considering that MDRPIs have a significant impact on patients’ quality of life, mortality, morbidity, and care costs, it is crucial to enhance the awareness of nurses working in high-risk units regarding MDRPI risk identification, monitoring, prevention, and care [3,19]. For all types of PIs, prevention is more effective and less costly than treatment. Therefore, nurses, who play a crucial role in preventing PIs, need to have sufficient knowledge to provide appropriate care. In this context, evaluating nurses’ knowledge of MDRPI prevention and assessing their current behaviors and attitudes towards preventive care are essential steps in identifying potential barriers. Such barriers may include a reluctance to adopt new evidence based practices, limited access to resources, excessive workload, insufficient equipment support, and the absence of a multidisciplinary team approach among nurses regarding pressure injury care [20,21]. Conceptually, MDRPI prevention depends on nurses’ knowledge and attitudes, which influence clinical decision-making and the consistent implementation of preventive practices in intensive care and surgical settings [19].

Previous studies have emphasized that nursing knowledge, adherence to preventive guidelines, and evidence-based practices play a critical role in pressure injury prevention, with nursing care identified as a central component of preventive strategies [19,21,22]. Determining nurses’ knowledge levels and attitudes related to PIs will contribute to the development of preventive care strategies. In order for a nurse to provide effective care, they must possess an adequate level of professional knowledge. The higher the level of knowledge, the higher the quality of care provided [21]. Studies have also indicated that in order for nurses to provide effective care for PIs, they need not only sufficient knowledge but also a positive attitude towards the subject [21,23].

The widespread use of technological devices, particularly in intensive care and surgical units, increases patients’ vulnerability to MDRPIs and requires a meticulous patient care process. Although the number of studies on MDRPIs has increased in recent years, research specifically evaluating the knowledge and attitudes of nurses working in intensive care and surgical units remains limited [3,17,24–29].Recent studies continue to report important knowledge and practice gaps among intensive care nurses regarding MDRPI prevention, highlighting the need for improved education and structured preventive approaches [19]. However, the existing literature generally focuses on a single clinical setting or determines prevalence and risk factors without simultaneously examining the knowledge and attitudes of nurses in high-risk units. Furthermore, the use of instruments with limited psychometric reporting may constrain the comparability and interpretability of findings. Therefore, this study aimed to assess the knowledge and attitudes of nurses working in intensive care and surgical units regarding MDRPIs and their prevention.

## Materials and methods

### Study design and setting

This cross-sectional study aimed to assess nurses’ knowledge and attitudes towards MDRPIs in intensive care and surgical units. The study was conducted between 15 November 2022 and 30 December 2022 at tertiary-level public hospital in Turkey. The target population comprised all 420 nurses working in the neurology, coronary, surgical, pediatric, and neonatal ICUs, as well as in the general surgery, neurosurgery, urology, and orthopedic services of the hospital.

### Participants

#### Inclusion and exclusion criteria.

Nurses eligible for inclusion in the study had to be actively working in the hospital's surgical units or ICUs during the study period, provide direct patient care, and voluntarily agree to participate by providing verbal and written informed consent. In addition, participants had to have been employed in their current unit for at least six months to ensure adequate clinical experience with medical device use.

Nurses were excluded from the study if they were administrative staff with no direct patient care responsibilities, were on leave for more than two weeks during the data collection period, or voluntarily declined to participate.

#### Study population and sample.

The target population consisted of all 420 nurses working in neurology, coronary, surgical, pediatric, and neonatal ICUs, as well as in general surgery, neurosurgery, urology, and orthopedic services of the hospital. The required sample size was calculated a priori using G*Power 3.1.9.4 [30]. Assuming a two-tailed test, a significance level of 0.05, 80% statistical power, and a medium effect size (Cohen’s d = 0.50, selected based on prior literature and Cohen’s conventional criteria for behavioral research rather than an arbitrary value), the minimum required sample size was calculated as 128 nurses. To account for potential non-response or incomplete data, the target sample size was increased by approximately 15%, and 150 nurses were ultimately included in the analysis. All eligible nurses were invited to participate, and 150 nurses were included in the final analysis (participation rate: 150/420, 35.7%). Recruitment and data collection were conducted during working hours in coordination with unit managers. Because participation was voluntary and dependent on nurses’ availability during shifts, the study reflects a non-probability (convenience/volunteer) sampling approach. Therefore, selection and non-response bias cannot be excluded. Detailed reasons for non-participation were not systematically recorded.

**Fig 1:**
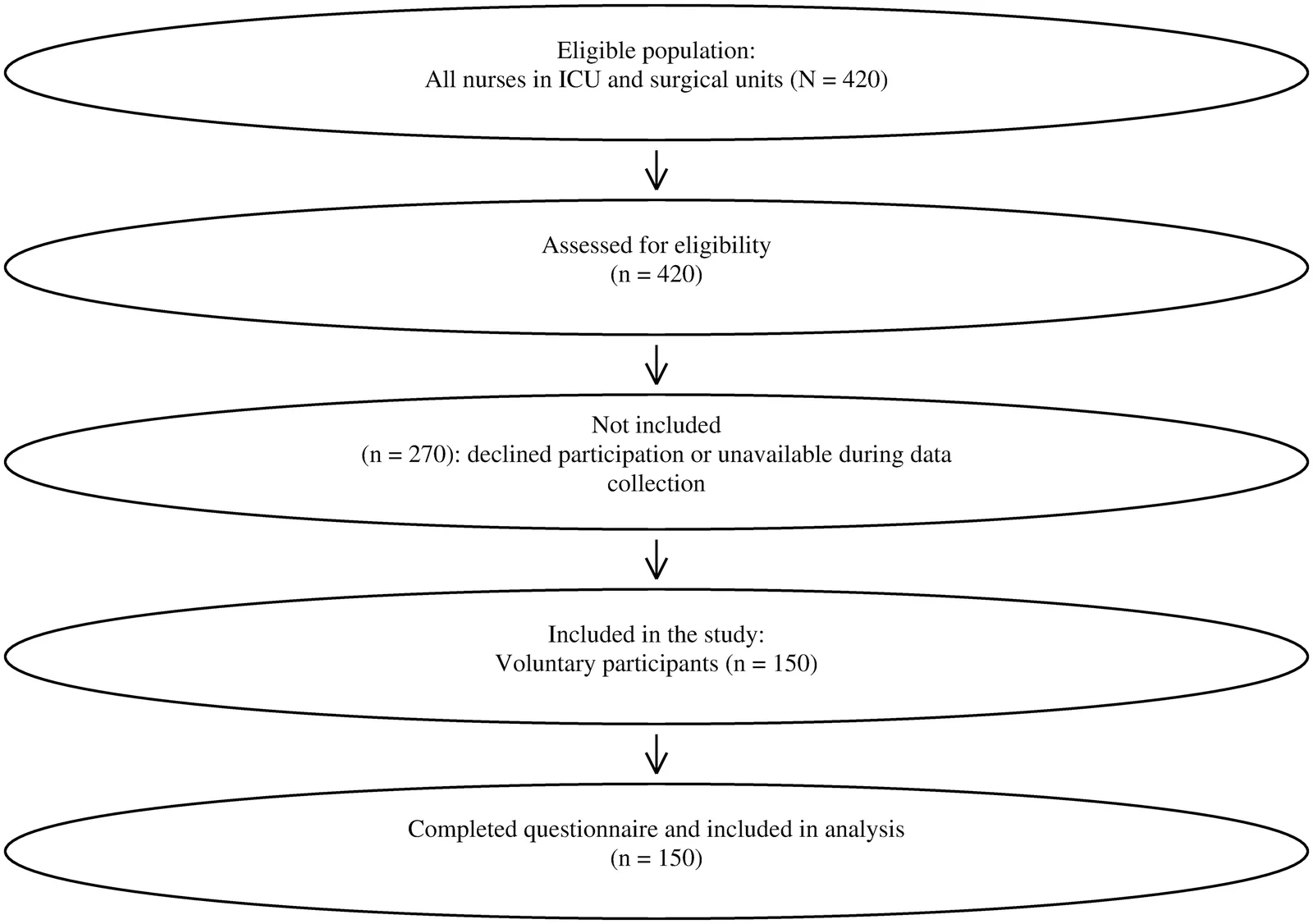
STROBE flow diagram of participant recruitment and inclusion in the study.

The STROBE flow chart of the study sample selection is presented in Fig 1.

### Data collection tools

The survey forms were collected from voluntarily participating nurses through face-to-face administration by the researchers during working hours. Where feasible, data collection was coordinated with unit managers to minimize disruption during shift changes and peak workload periods. The approximate time for completing the forms was 15–20 minutes per nurse. All relevant data are within the manuscript and its Supporting Information files (S1 Table and S2 Data). The detailed item-level results are provided as S1 Table, and the de-identified dataset underlying the results is provided as S2 Data.

### Nurse identification form

The form includes a total of 8 questions concerning the nurses’ sociodemographic characteristics such as age, gender, educational status, and the unit they work in.

### Medical Device-Related Pressure Injury Knowledge and Attitude Questionnaire (MDRPIKAQ) form

MDRPIKAQ developed by researchers based on relevant literature [11,15,22,24,25,31–35] consists of a total of 52 items, including 45 related to nurses’ knowledge and 7 related to attitudes towards MDRPIs.

The development of the MDRPIKAQ followed a structured multi-stage process. First, a comprehensive review of the relevant literature was conducted to identify the conceptual domains of MDRPI prevention. Based on this review, the co-authors collaboratively generated an initial pool of items through multiple structured meetings held both online and face-to-face to ensure content coverage, conceptual clarity, and clinical relevance.

The preliminary draft of the questionnaire was subsequently submitted to a panel of nine experts, including two wound care nurses and seven nursing faculty members with expertise in pressure injury prevention. Experts evaluated each item in terms of relevance, clarity, and content adequacy using a structured expert evaluation form based on the Davis technique. Written feedback from the experts was systematically reviewed, and the questionnaire was revised through consensus among the co-authors before finalization.

The knowledge section of the MDRPIKAQ is divided into three sub-dimensions: description sub-dimension with 4 items, risk assessment sub-dimension with 21 items, and prevention and care sub-dimension with 20 items. Each statement in the knowledge section can be answered as “true,” “false,” or “I don't know.” Each correct answer is scored as “1,” while incorrect and “I don't know” answers are scored as “0.” Out of the items in this section, 28 are arranged to be answered as “false,” and 17 are arranged to be answered as “true.” A score ranging from “0–45” can be obtained from the knowledge section of the questionnaire.

The attitude section of the questionnaire consists of seven questions. These items are rated on a five-point Likert scale, where the evaluation of the first six items is as follows: “strongly agree (1 point),” “agree (2 points),” “neutral (3 points),” “disagree (4 points),” and “strongly disagree (5 points).” The 7^th^ item in the questionnaire is reverse coded for scoring purposes. A score ranging from “7–35” can be obtained from the attitude section.

Higher scores on the knowledge and attitude sections indicate greater knowledge levels and more positive attitudes toward MDRPI prevention. The total score that can be obtained from the questionnaire ranges from a minimum of “7” to a maximum of “80.” The cutoff value for the questionnaire was determined as “45 points” using the Angoff Method.

According to the Angoff method, a standard-setting procedure based on expert judgment, the cutoff score for the MDRPIKAQ was set at 45 points. For the standard-setting procedure, the expert panel was provided with written instructions describing a nurse meeting the minimum acceptable competency level in intensive care or surgical settings. The concept of a ‘minimally competent nurse’ was clarified, and sample items were reviewed to ensure panel members had a common understanding of the rating criteria. Experts independently estimated the probability that such a nurse would correctly answer each knowledge item and demonstrate an appropriate attitude response. A single rating round was conducted and the estimated probabilities were averaged across experts, and the summed mean values across all items constituted the final minimum competency threshold. Scores below the cut-off of 45 indicate that a participant’s overall performance is below the minimum competency threshold, rather than reflecting only insufficient knowledge or negative attitudes. Knowledge and attitude were scored separately, but no separate cut-offs were set for these sections, because the Angoff method was applied to the total score. This ensures that the total score reflects overall competency in knowledge and attitudes toward MDRPI prevention. Participants scoring ≥45 were considered to demonstrate sufficient overall competency in knowledge and attitudes toward MDRPI prevention. Scores ≤44 were interpreted as reflecting performance below the minimum acceptable competency threshold defined by expert judgment. Because the total score represents a composite measure, classification below the cutoff indicates an overall level below the expected standard rather than exclusively negative attitudes or insufficient knowledge in isolation.

The MDRPIKAQ demonstrated acceptable reliability and validity, with Cronbach’s alpha values of 0.770 (knowledge) and 0.707 (attitude), and a CVI of 0.97, confirming the instrument’s suitability for assessing nurses’ knowledge and attitudes toward MDRPIs.

To assess the content validity of the questionnaire, the opinions of nine experts were sought, including two wound care nurses and seven nursing faculty members. The content validity of the questionnaire was assessed using the Davis technique. The Content Validity Index (CVI), calculated from expert ratings, was found to be 0.97, indicating excellent content validity. Kendall's W = 0.294, indicating a moderate level of agreement among experts (p < 0.001). Concordance analysis using Kendall’s W was performed to assess agreement among experts during the content validity evaluation, providing a measure of consistency in their ratings across questionnaire items.The internal consistency reliability of the MDRPIKAQ was assessed using Cronbach's Alpha coefficient. The Cronbach's Alpha was found to be 0.770 for the knowledge section and 0.707 for the attitude section, indicating acceptable internal consistency reliability for both parts of the questionnaire.

A pilot study was conducted with 10 nurses to confirm the comprehensibility of the statements in the questionnaire. The pilot participants were not included in the final study sample. The pilot study findings indicated that no modifications to the questionnaire were necessary, thereby confirming the clarity and comprehensibility of the instrument.

### Statistical analysis

Data analysis was conducted using SPSS 24.0 statistical software. The distributions of the data scores were tested for normality using the Kolmogorov-Smirnov and Shapiro-Wilk tests. The results showed that all variables were approximately normally distributed (Kolmogorov-Smirnov p > 0.05; Shapiro-Wilk p > 0.05).

We used descriptive tests, one-way analysis of variance (ANOVA), independent samples t-test, and Pearson correlation analyses were applied, since all variables were approximately normally distributed. Concordance analysis and linear regression analyses were also performed to evaluate the data. Two multiple linear regression analyses were performed to identify predictors of MDRPIKAQ knowledge and attitude scores. The dependent variables were the total knowledge score and total attitude score, respectively. Prior to the analyses, the assumptions of linear regression (linearity, normality of residuals, homoscedasticity, and absence of multicollinearity) were checked and no issues were detected. A significance level of p < 0.05 was considered statistically significant. No formal correction for multiple testing was applied because the regression analyses were limited in number and specified to address predefined study questions rather than broad exploratory screening.

### Ethical considerations

This study was approved by the Ethics Committee of the University (Decision Number: 2022/301) and the hospital's chief physician where the study was conducted. After providing the necessary explanations to the participants, verbal and written consent were obtained. The study was conducted in accordance with the principles of the Helsinki Declaration.

## Results

### Demographic and professional characteristics of the participants

The mean age of the 150 nurses participating in the study was determined to be 33.07 years (±6.31 years), and the mean years of service was found to be 10.07 years (±6.49 years).

As summarized in Table 1, the majority of nurses held a bachelor’s degree and worked in adult ICUs. Most participants reported not having received prior training on MDRPI prevention, yet a high proportion expressed willingness to receive such training. In addition, the majority indicated a perceived need for a nursing care protocol for MDRPI prevention.

**Table 1:** Nurses’ sociodemographic and professional characteristics.

Variables	Mean ± SD	Median (min – max)
**Age**	33.07 ± 6.31	23-47
**Work Experience (years)**	10.07 ± 6.49	1-31
	**n**	**%**
**Age Categories**		
18-29	50	33.3
30-39	71	47.3
40-49	29	19.3
**Sex**		
Women	91	60.7
Men	59	39.3
**Graduated Nursing Program**		
High School*	8	5.3
Bachelor’s degree**	132	88.0
Postgraduate degree ***	10	6.7
**Working Department**		
Adult ICU	76	50.7
Newborn ICU	18	12.0
Child ICU	18	12.0
Surgical services	38	25.3
**Received MDRPI Training**		
No	108	72.0
Yes	42	28.0
**Willingness to Receive MDRPI Training**		
Yes	118	78.6
No	32	21.4
**Need for Nursing Care Protocol**		
Yes	124	82.7
No	26	17.3
**Medical Devices Most Commonly Causing MDRPIs**		
Endotracheal Tube	21	14.0
Noninvasive Mask	110	73.3
Urinary Catheter	17	11.3
Nasogastric Tubes	2	1.4

**Abbreviations:** SD = Standard Deviation, MDRPIs = Medical Device-Related Pressure Injuries

*: High school is four years long

**: Bachelor’s degree is 4 years long

***: Postgraduate degree is 2 years long

As shown in Table 2, the MDRPIKAQ knowledge sub-dimensions yielded mean scores of 2.07 ± 0.68 for description, 12.06 ± 3.74 for risk factors, and 9.65 ± 2.60 for prevention and care. Overall knowledge levels were moderate, with a total score ranging from 0 to 45 (mean: 24.33 ± 5.20).

**Table 2:** Knowledge and attitude scores regarding MDRPIs.

MDRPIKAQ’ s Sub-Dimension	Mean ± SD	Min-Max
Description	2.07 ± 0.68	0-4
Risk Factors	12.06 ± 3.74	0-21
Prevention and Treatment	9.65 ± 2.60	0-20
Knowledge Total Scores	24.33 ± 5.20	0-45
Attitude Total Scores	16.64 ± 3.48	7-35

Abbreviations: SD = Standard Deviation, Min = Minimum, Max = Maximum,

Attitude scores ranged from 7 to 35 (mean: 16.64 ± 3.48). Using the Angoff method, a cutoff score of 45 was applied to classify sufficient overall knowledge and attitude; only 36.7% of nurses met or exceeded this threshold (Table 2).

Detailed item-level distributions for all knowledge and attitude statements are provided in S1 Table to avoid repetition in the main text. Briefly, nurses demonstrated high correct response rates for core MDRPI concepts (e.g., device-shaped injury patterns and the need to inspect skin under immobilizing devices), whereas lower correct response rates were observed for items related to perineal skin assessment in patients with Foley catheters, differentiating MDRPIs from immobility-related PIs, and temperature-related risk factors (S1 Table). At the attitude-item level, notable misconceptions were observed, including agreement that MDRPI prevention is difficult, that protocols are unnecessary, and that prevention is primarily a physician’s responsibility (S1 Table). There were statistically significant differences between nurses’ MDRPIKAQ knowledge scores and age. A post hoc Tukey test showed that the difference in knowledge scores was due to nurses aged 40–49 years. Similarly, there were statistically significant differences between nurses’ MDRPIKAQ attitude scores and gender, educational status, and unit in which they worked (p < .05; Table 3). Post hoc analysis indicated that the difference in attitude scores was associated with nurses who held a bachelor's degree.

**Table 3:** Nurses’ average knowledge and attitude total scores according to their demographic and professional characteristics.

Variables	Knowledge Score Mean ± SD	Variance or t Test and P values	Attitude Score mean ± SD	Variance or t Test and P values
**Age**				
20-29 Years of age	23.46 ± 6.71	F = 3.21	16.90 ± 4.23	F = 0.22 p = .804
30-39 Years of age	24.08 ± 4.73	p = .043*	16.50 ± 3.23	
40-49 Years of age	26.44 ± 2.32		16.48 ± 2.54	
**Gender**				
Women	24.20 ± 5.42	t = 0.72	16.07 ± 3.34	t = 2.48 p= .01*
Men	24.52 ± 5.01	p = .79	17.49 ± 3.53	
**Education**				
High school	26.75 ± 2.25	F = 0.93	14.87 ± 2.79	F = 3.86 p= .02**
Bachelor’s Degree	24.22 ± 5.44	p = .399	16.91 ± 3.43	
Post-graduate degree	23.80 ± 3.91		14.30 ± 3.56	
**Department of expertise**				
Adult ICU	24.22 ± 5.70	F = 0.78	16.86 ± 5.62	F = 1.83 p= .04*
Newborn ICU	24.83 ± 3.80	p = .97	17.38 ± 3.46	
Child ICU	24.11 ± 5.64		15.68 ± 3.92	
Surgery Services	24.42 ± 4.85		14.44 ± 2.38	
**Years of experience**				
1-5 year	23.55 ± 6.64	F = 2.1	17.43 ± 3.96	F = 1.44 p = .23
6-10 year	23.12 ± 5.93	p = .10	16.35 ± 3.51	
11-16 year	25.48 ± 2.90		16.34 ± 3.08	
17-25 year	25.91 ± 2.53		15.58 ± 2.39	
**Training on Pressure Injury**				
Yes	25.08 ± 4.16	t = 2.87	16.62 ± 3.47	t = −0.07 p = .94
No	22.40 ± 7.03	*p*= .005*	16.66 ± 3.51	

Abbreviations: SD = Standard Deviation, F = Variance Analysis (ANOVA), t = Independent t-Test,

*p < .05, **p < .01

Pearson correlation analysis showed statistically significant positive correlations between the MDRPIKAQ knowledge sub-dimensions and total knowledge scores, as well as selected associations with total attitude scores (Table 4). Specifically, the diagnosis sub-dimension was positively correlated with the prevention and care sub-dimension (r = 0.251, p = 0.002) and with total knowledge score (r = 0.299, p < 0.001). The risk assessment sub-dimension was strongly correlated with the total knowledge score (r = 0.850, p < 0.001). The prevention and care sub-dimension was also positively correlated with total knowledge (r = 0.715, p < 0.001) and total attitude scores (r = 0.213, p = 0.009). No statistically significant correlation was observed between total knowledge and total attitude scores (r = 0.034). The full correlation matrix is presented in Table 4.

**Table 4:** Correlation analysis of MDRPIKAQ sub-dimensions and total scores.

Variable	Diagnosis	Risk Assessment	Prevention & Care	Attitude Total	Knowledge Total
Diagnosis	1	0.061	0.251**	0.143	0.299**
Risk Assessment	0.061	1	0.262**	−0.129	0.850**
Prevention & Care	0.251**	0.262**	1	0.213**	0.715**
Attitude Total	0.143	−0.129	0.213**	1	0.034
Knowledge Total	0.299**	0.850**	0.715**	0.034	1

Based on these findings, separate regression models were developed to identify independent predictors of knowledge and attitude while avoiding reciprocal modelling between the two constructs (Table 5). In the model predicting Knowledge Total Score, receipt of MDRPI-related education remained the only significant predictor. The overall model was statistically significant, F(1,148) = 5.81, p = .005, explaining 7.3% of the variance in knowledge score (R² = .073; adjusted R² = .061). Participants who had received MDRPI-related education demonstrated significantly higher knowledge scores than those who had not received such education (B = 2.679, SE = 0.932, 95% CI [0.837, 4.521], p = .005). Multicollinearity diagnostics indicated no evidence of collinearity (VIF = 1.00).

**Table 5:** Multiple linear regression models for MDRPIKAQ knowledge and attitude scores.

Predictor	B	SE	95% CI	p	VIF
**Model 1. Outcome: Knowledge Total Score**					
Intercept	22.405	0.791	[20.842, 23.968]	<.001	—
Received MDRPI education (Yes vs No†)	2.679	0.932	[0.837, 4.521]	.005	1.000
**Model fit statistics**					
R² = .073	Adjusted R² = .061	F(1,148)=5.81	p = .005		
**Model 2. Outcome: Attitude Total Score**					
Intercept	15.184	0.538	[14.121, 16.247]	<.001	—
Adult ICU vs Surgical clinics‡	2.429	0.660	[1.124, 3.734]	<.001	1.000
Neonatal ICU vs Surgical clinics	−0.129	0.949	[−2.004, 1.746]	.892	1.000
Pediatric ICU vs Surgical clinics	2.094	0.949	[0.218, 3.969]	.029	1.000
**Model fit statistics**					
R² = .068	Adjusted R² = .062	F(1,147)=10.73	p = .001		

**Note.** B = unstandardized regression coefficient; SE = standard error; CI = confidence interval; VIF = variance inflation factor. † Reference category: no prior MDRPI-related education. ‡ Department was entered as a categorical variable using dummy coding. Reference category: Surgical clinics.

A second regression model was constructed to evaluate factors independently associated with Attitude Total Score (Table 5). Department was entered as a categorical variable using surgical clinics as the reference category. The final model reached statistical significance, F(1,147) = 10.73, p = .001, accounting for 6.8% of the variability in attitude scores (R² = .068; adjusted R² = .062). Compared with healthcare professionals working in surgical clinics, those employed in Adult Intensive Care Units reported significantly higher attitude scores (B = 2.429, SE = 0.660, 95% CI [1.124, 3.734], p < .001). Similarly, participants working in Pediatric Intensive Care Units demonstrated higher attitude scores (B = 2.094, SE = 0.949, 95% CI [0.218, 3.969], p = .029). No statistically significant difference was observed between Neonatal Intensive Care Units and surgical clinics (B = −0.129, SE = 0.949, 95% CI [−2.004, 1.746], p = .892).

To further evaluate whether the absence of association between knowledge and attitude was influenced by participant characteristics, an additional partial correlation analysis was performed controlling for work experience, receipt of MDRPI-related education, and department (Table 6). The initial non-significant association between Knowledge Total Score and Attitude Total Score remained unchanged after adjustment (partial r = .045, p = .590). The minimal change in correlation magnitude after controlling for these variables suggests that the lack of association between knowledge and attitude was not explained by differences in educational exposure, professional experience, or clinical setting. These findings support treating knowledge and attitude as related but analytically distinct constructs and further justify modelling them as separate outcomes.

**Table 6:** Partial correlation between knowledge total score and attitude total score controlling for selected covariates.

Variables	Zero-order correlation (r)	Partial correlation (r)	p
Knowledge Total Score – Attitude Total Score	0.034	0.045	0.590

**Note.** Partial correlation was adjusted for work experience, receipt of MDRPI-related education, and department. Zero-order correlations represent unadjusted Pearson correlation coefficients. Partial correlation coefficients represent the association between Knowledge Total Score and Attitude Total Score after controlling for the specified covariates.

## Discussion

In ICUs and surgical units, where medical devices are frequently used, nurses play an important role in preventing or reducing MDRPIs. It is crucial for nurses to have the necessary level of knowledge and positive attitudes towards the prevention of MDRPIs in order to provide effective care [37]. In this study, nurses demonstrated moderate knowledge (24.33 ± 5.2/45) and moderate attitudes (16.64 ± 3.48/35) toward MDRPI prevention. Sub-dimension analysis based on the MDRPIKAQ revealed that knowledge levels were comparatively higher in the description (2.07 ± 0.68 / 4) and risk factors (12.06 ± 3.74 / 21) domains, while scores in the prevention and care sub-dimension remained lower (9.65 ± 2.60 / 20). This pattern is important because it implies that “knowing” MDRPI concepts and risk factors may not automatically translate into “doing” the routine preventive steps consistently at the bedside especially in settings with frequent device use and high workload. A review of the extant literature revealed a paucity of studies addressing knowledge and attitudes regarding MDRPI, particularly among ICU and surgical nurses. Taken together, these findings suggest that moderate knowledge levels may represent a common pattern across different clinical settings rather than an institution specific issue [25,37]. At the same time, direct comparisons should be made cautiously because differences in staffing patterns, scope of nursing practice, access to wound care support, and the presence (or absence) of unit based protocols can meaningfully shape both learning opportunities and daily prevention behaviours. Only 36.7% of nurses met the Angoff-based cutoff (≥45/80). This finding suggests that having a university degree alone may not be sufficient to ensure adequate preparedness for MDRPI prevention and that additional structured education may be beneficial. Kim et al. (2019) reported an association between participation in pressure injury–related training and higher prevention performance among nurses [18]. Similarly, studies by Sonmez and Bahar (2022), Wei et al. (2020), and Fu et al. (2023) highlighted that ICU nurses often have inadequate knowledge and attitudes toward MDRPIs [26,27,34] Rather than simply repeating that “knowledge is low,” these studies together with our findings suggest a more practical issue: preventive care steps (such as when and where to check the skin, and how to act early) may not be embedded as routine practice unless education is reinforced by clear protocols and unit-level expectations. Another finding from the study is that the majority of the nurses did not receive MDRPIs education (72%), but three quarters of them expressed a desire to receive education on this topic. This combination indicates an unmet educational need and a receptive climate for MDRPI-focused in-service programs accordingly, improving access to structured training and clear unit-level protocols may support consistent bedside prevention practices, particularly in high-workload settings [34].

In our study, the questionnaire assessing nurses’ knowledge and attitudes towards MDRPIs included the question “*The formation of MDRPIs is different from PIs caused by immobility*” which was designed to assess their knowledge of staging. Only a small minority of the participants (less than one-fifth) answered this question correctly, indicating a limited understanding of the differences in etiology between MDRPIs and immobilizing PIs. Similar to the findings of our study, Erbay et al (2019) conducted a study with 122 ICU nurses to assess their knowledge level about MDRPIs and reported that they received the highest scores on skin assessments for staging and the lowest scores on mucosal assessments [37]. This alignment is clinically meaningful: if MDRPIs are viewed as “the same” as immobility-related PIs, nurses may be uncertain about how to stage, where to assess, and which device-specific precautions are required [17]. When examining the percentage distribution of nurses’ responses to the items in the knowledge section, the items with the lowest correct response rates were those addressing the distinction between MDRPIs and immobility-related PIs, the impact of perioperative temperature regulation on MDRPI risk, and the recommended frequency of perineal skin assessment in patients with Foley catheters. For all of these items, correct responses were provided by only a small proportion of nurses, indicating marked knowledge gaps in staging, temperature-related risk factors, and the timing of skin assessments (see S1 Table). Studies similar to our findings have been conducted [24,31,34,36]. For instance, Fu et al. (2023) reported that ICU nurses demonstrated insufficient knowledge regarding MDRPI, particularly in the domains of concept, staging, and skin assessment [27]. Similarly, Sonmez and Bahar found that nurses who had received specific training on MDRPIs achieved significantly higher knowledge scores compared with those who had not received such training [34]. Kim et al. found that nurses received high baseline knowledge, and participation in pressure injury related training was associated with higher prevention performance among nurses [18]. However, in the same study, nurses received high scores for assessing patients’ skin conditions but lower scores regarding the frequency of care. They scored lowest on the item related to assessing the perineal area and surrounding skin in patients with a foley catheter. Taken together, these patterns suggest that nurses can often recognise “high-risk devices” yet may be less confident about “how often” and “which specific sites” should be assessed once the device is in place an important gap for prevention. These findings highlight the need for educational content specifically focused on staging, device-related pathophysiology, and evidence-based intervals for skin assessment in patients with medical devices. Surgical patients are at high risk for developing PIs due to several factors before, during and after surgery. Studies have been conducted to identify the factors that contribute to PIs during the surgical process [5,8]. For example, Munro et al (2010) reported that intraoperative hypothermia may lead to circulatory disturbances, while hyperthermia may help maintain skin hydration and reduce the risk of pressure injury [5]. In our study, only about one-fifth of participants answered correctly the statement “*MDRPIs reduce the risk of developing pressure injury with hypothermia, while hyperthermia increases the risk of medical pressure injury*” indicating a significant lack of knowledge in understanding temperature-related risk factors associated with the development of MDRPIs.

According to the literature, patients undergoing surgical procedures are at risk for developing PIs, particularly during prolonged operations and when medical devices are used intraoperatively [2,26]. In order to prevent the development of PIs in patients undergoing surgery, risk assessment should be a priority. In our study, approximately half of the nurses agreed with the statement “*The same measurement tools are used for MDRPIs and immobilization related* PIs *in surgical patients*,” indicating possible confusion regarding the tools used for assessment. Furthermore, the item “*The occurrence of MDRPIs is different from* PIs *due to immobilization*” received correct responses from only a minority of nurses, reflecting a low level of differentiation in clinical understanding of the two types of injury. Such conceptual confusion may contribute to under-recognition of device-related risks and missed opportunities for early protective actions, as device-specific monitoring and prevention strategies have been emphasized in previous literature [37].

Practically, this may lead to under-recognition of device-related risk and missed opportunities for early protective actions (e.g., repositioning the device, using protective dressings, and scheduled inspection). One of the highest correct response rates in the knowledge section of our study (nearly nine out of ten nurses) was observed for the statement, “*Patients who are unable to achieve glycemic control regulation in the preoperative period are at risk for medical pressure injury*. " This high rate may indicate that nurses are aware of the relationship between surgical stress and impaired glycemic control. In this context, these results may reflect a level of clinical awareness that contributes to the recognition of metabolic risk factors associated with the development of MDRPIs. However, previous research has highlighted that awareness alone may not consistently translate into preventive performance without institutional reinforcement and structured education [18,31]. In our study, nurses also frequently reported encountering MDRPIs caused by non-invasive oxygen masks (73.3%), endotracheal tubes (14.0%), and urinary catheters (11.3%), which are among the devices that contribute to MDRPIs. Similarly, Black and Kalowes (2016) reported in their study that MDRPIs are likely caused by devices related to oxygen administration and airway management [11]. In our study, when examining the distribution of items in the questionnaire, it was found that the majority of nurses (94%) answered the question “*There is no need to check the skin condition of a patient with an endotracheal tube*” correctly. This consistency is encouraging, because it suggests that nurses may be more confident with commonly encountered airway-related devices yet the earlier item-level gaps indicate that confidence may not extend equally to other device sites and assessment intervals. The nurses’ responses regarding frequently encountered medical devices and their correct responses regarding the need to check the skin condition of a patient with an endotracheal tube are mutually supportive and consistent with the literature [3,14,15,17,24,28,34].

When examining the attitudes section of the questionnaire, the overall mean score suggested a relatively positive attitude toward MDRPI prevention; however, item level analysis revealed several important misconceptions. Many nurses agreed with statements such as “MDRPIs heal on their own” and “Preventing MDRPIs is difficult”, and a considerable proportion also agreed that “Preventing MDRPIs is not a priority for nurses” and that “MDRPIs are less important than other PIs”. Similar knowledge–attitude inconsistencies have been reported in previous MDRPIs studies, suggesting that misconceptions may negatively influence preventive behaviors [18,31]. These misconceptions may function as practical barriers, as they may reduce the urgency of early skin inspection, delay protective interventions, and contribute to deprioritising MDRPIs prevention during busy shifts. Rather than attributing these beliefs solely to individual factors, previous studies have emphasized the importance of clear institutional protocols and structured educational support in shaping preventive practices [35]. Therefore, interventions should not only provide knowledge, but also clarify accountability (nurse-led prevention), and normalise MDRPI checks as a routine part of device care.

In this study, statistically significant differences in knowledge scores were observed by age, whereas attitude scores differed by gender, education level, and specialty (Table 3). In the multivariable regression analyses, however, receipt of MDRPI-related education emerged as the only independent predictor of higher knowledge scores. For attitude, department was the only independent predictor, with nurses working in Adult and Pediatric Intensive Care Units demonstrating more favorable attitude scores than those working in surgical clinics. In addition, no statistically significant zero-order or adjusted association was observed between total knowledge and total attitude scores. These findings suggest that knowledge and attitude should be interpreted as related but analytically distinct constructs, and that targeted in-service education and workplace context may influence different aspects of MDRPI prevention. Consistent with our findings, Zhang et al. (2021) reported that knowledge scores regarding MDRPIs improved with increasing age and professional experience [25]. Tan et al. (2020) also emphasized the importance of integrating theoretical education with practical application in nurses’ understanding of MDRPI prevention [29]. Similarly, Nuru et al. (2015), Kim et al. (2019), and Zhang et al. (2021) reported that higher levels of education and greater professional experience were associated with better knowledge and preventive practices regarding pressure injuries [2,18,38]. Taken together, these findings support the value of structured pre-service and in-service training that is closely linked to daily practice and reinforced by clear unit-level protocols to strengthen MDRPI prevention.

### Limitations of the study

Our study was conducted in a single center, included only nurses working in surgical and ICUs, and involved a relatively limited sample. A non-probability sampling approach, specifically convenience sampling, was used, as participation was voluntary and not based on random selection. Therefore, the results may not reflect the knowledge and attitudes of all nurses regarding MDRPI in Turkey or in other clinical specialties. Although comparable to ICU-based surveys, the participation rate may still introduce response bias, as nurses who chose to participate may differ systematically from those who did not. In addition, because the study was conducted in a single institution and included a limited number of specialty areas, differences in institutional protocols, educational opportunities, and clinical practices across settings may limit the generalizability of the findings.

In addition, the cross-sectional design does not allow for causal inferences about the relationships between knowledge, attitudes, and associated factors. Because all measurements were taken at one time point, we cannot determine whether one factor led to another (i.e., we cannot establish “cause and effect” or the direction of the relationship). Even though we used multivariable regression to identify independent predictors, these findings should still be interpreted as associations, and unmeasured factors may have influenced the results.

Self-report data may be influenced by a tendency to give professionally ‘expected’ answers (social desirability). Some participants may have chosen answers that seemed professionally appropriate rather than reflecting their usual knowledge or attitudes, which may have affected the accuracy of the reported scores.

Future multi-center studies with larger and more diverse samples and longitudinal or interventional designs are needed to confirm and extend these findings.

## Conclusion

In this study, nurses demonstrated moderate levels of knowledge and attitudes toward the prevention of MDRPIs. Overall scores indicated a generally positive orientation, while some item-level analyses suggested misconceptions regarding the preventability, relative importance, and perceived responsibility for MDRPIs. Knowledge scores varied significantly by age, whereas attitude scores differed by gender, level of education, and unit of practice. No statistically significant association was observed between knowledge and attitude scores. In the regression analyses, receipt of MDRPI-specific education was associated with higher knowledge scores, whereas working in Adult and Pediatric Intensive Care Units was associated with more favorable attitude scores.

These findings support the integration of MDRPI-specific content into nursing curricula and emphasize the use of interactive educational strategies such as simulation and case-based learning to strengthen both knowledge and attitudes. Future multicenter studies are recommended to enhance generalizability. In addition, longitudinal and observational studies, as well as mixed-method designs that combine quantitative assessment with qualitative exploration of nurses’ experiences and perceived barriers, are recommended to evaluate the long-term impact of educational and organizational interventions on MDRPI prevention in clinical practice.

## Supporting information

S1 Table Item-level distribution of nurses’ responses to all MDRPIKAQ knowledge and attitude items. (DOCX)

S2 Data De-identified raw data for nurses’ MDRPIKAQ knowledge and attitude scores and related study variables. (CSV)

S3 Codebook Variable definitions and scoring information for the de-identified dataset. (DOCX)

S4 Checklist STROBE checklist for cross-sectional studies. (DOCX)
