# Supplementary material for: Nurses' knowledge and attitudes towards medical device-related pressure injuries in intensive care and surgical units: Cross-sectional study
Source: PLoS One. 2026 Aug 19;21(8):e0356550. doi: 10.1371/journal.pone.0356550 (PMC13489443; doi:10.1371/journal.pone.0356550)
Supplement: Supplementary file 1 [file pone.0356550.s001.DOCX]

**S1 Table. Item-level distribution of nurses’ correct responses in the MDRPIKAQ knowledge section and response distribution in the attitude section.**

| **Statements Regarding Knowledge** | | | | | | | | | | **Correct responses*** | | | | |
| --- | --- | --- | --- | --- | --- | --- | --- | --- | --- | --- | --- | --- | --- | --- |
|  |  |  |  |  |  |  |  |  |  | **n** | | **%** | | |
| 1 | MDRPIs tend to take the shape of the device or instrument (T) | | | | | | | | | 136 | | 90.7 | | |
| 2 | MDRPIs is seen in the superficial epidermal layer and does not progress to the lower layers. (F) | | | | | | | | | 119 | | 79.3 | | |
| 3 | The formation of MDRPIs are different from pressure injuries caused by immobility (F) | | | | | | | | | 24 | | 16.0 | | |
| 4 | MDRPIs are simple. minor injuries. requiring no further treatment (F) | | | | | | | | | 132 | | 88.0 | | |
| **Sub-Dimension 2-Risk Factors** | | | | | | | | | | | | | | |
| 5 | All age groups using medical devices have the same risk of medical pressure injury in patients (F) | | | | | | | | | 45 | | 30.0 | | |
| 6 | MDRPIs are more common in chronic patients with circulatory disorders (T) | | | | | | | | | 131 | | 87.3 | | |
| 7 | Patients who cannot achieve glycemic control regulation in the preoperative period are at risk of medical pressure injury (T) | | | | | | | | | 133 | | 88.7 | | |
| 8 | Devices used only to support breathing cause medical pressure injury (F) | | | | | | | | | 125 | | 83.3 | | |
| 9 | Patients with no feeding problems have less risk of medical pressure injury (T) | | | | | | | | | 54 | | 36.0 | | |
| 10 | The same measurement tools are used for MDRPIs and immobilization-related pressure injuries in surgical patients (F) | | | | | | | | | 72 | | 48.0 | | |
| 11 | The risk of pressure injury in patients with edema is higher than in other patient groups (T) | | | | | | | | | 141 | | 94.0 | | |
| 12 | The type of anesthesia (general and regional) administered to surgical patients does not affect the risk of medical device-related pressure injuries (F) | | | | | | | | | 60 | | 40.0 | | |
| 13 | The duration of surgery affects MDRPIs. The risk of pressure injury is low in operations that are not longer than 2 hours. (T) | | | | | | | | | 117 | | 78.0 | | |
| 14 | The risk of MDRPIs are the same in surgical patient positions given in surgeries (F) | | | | | | | | | 63 | | 42.0 | | |
| 15 | Cachectic and obese patients are at higher risk of MDRPIs(T) | | | | | | | | | 137 | | 91.3 | | |
| 16 | Regardless of all concomitant diseases and factors, the risk of developing MDRPIs are the same in all surgeries (T) | | | | | | | | | 35 | | 23.3 | | |
| 17 | Smoking increases the risk of MDRPIs (T) | | | | | | | | | 116 | | 77.3 | | |
| 18 | MDRPIs reduce the risk of pressure injury with hypothermia while hyperthermia increases the risk of MDRPIs (F) | | | | | | | | | 31 | | 20.7 | | |
| 19 | Heating devices applied to the patient during awakening may cause MDRPIs (T) | | | | | | | | | 121 | | 80.7 | | |
| 20 | Fluid loss during and after surgery does not increase the risk of developing MDRPIs (F) | | | | | | | | | 98 | | 65.3 | | |
| 21 | Improper use of compression stockings is not a risk for medical pressure sores (F) | | | | | | | | | 129 | | 86.0 | | |
| 22 | There is no risk of pressure injury for the patient whose total time from the pre-operative service to the postoperative service is up to 2 hours. (F) | | | | | | | | | 50 | | 33.3 | | |
| 23 | The patient whose preoperative fasting time is less than 12 hours has no risk of MDRPIs (F) | | | | | | | | | 63 | | 42.0 | | |
| 24 | The size of medical devices and equipment does not vary according to the patient (F) | | | | | | | | | 142 | | 94.7 | | |
| 25 | Selecting medical devices from soft materials reduces the risk of MDRPIs development (T) | | | | | | | | | 134 | | 89.3 | | |
| **Sub-Dimension 3-Prevention and Treatment** | | | | | | | | | | | | | | |
| 26 | The skin under the medical device should be observed once a day. (F) | | | | | | | | | 108 | | 72.0 | | |
| 27 | If a pressure sore occurred, the area can be massaged to increase circulation (F) | | | | | | | | | 99 | | 66.0 | | |
| 28 | A protein-rich diet is sufficient for MDRPIs to occur (F) | | | | | | | | | 120 | | 80.0 | | |
| 29 | Fluid should be restricted to limit edema in the pressure sore. (F) | | | | | | | | | 121 | | 80.7 | | |
| 30 | Tight fixation of the medical device to the skin surface may increase the risk of MDRPIs (T) | | | | | | | | | 127 | | 84.7 | | |
| 31 | Repositioning should be avoided to prevent dislocation of the medical device (F) | | | | | | | | | 125 | | 83.3 | | |
| 32 | No materials should be used to fix the medical device (F) | | | | | | | | | 130 | | 86.7 | | |
| 33 | It should be ensured that the skin under the medical device is kept moist (F) | | | | | | | | | 120 | | 80.0 | | |
| 34 | The skin condition of a patient with splints, traction, or cervical collars should be checked (T) | | | | | | | | | 143 | | 95.3 | | |
| 35 | The varicose stockings, elastic bandages, or pneumatic compression devices should be checked for the condition of the skin on the feet, thighs, and calves (T) | | | | | | | | | 130 | | 86.7 | | |
| 36 | Cervical sleeves should be replaced with a soft collar during acute care (T) | | | | | | | | | 128 | | 85.3 | | |
| 37 | The perineal area and its surroundings of a patient with a foley catheter should be checked twice a day for pressure injury (F) | | | | | | | | | 14 | | 9.3 | | |
| 38 | The skin condition of the patient with arterial catheter, hemodialysis and ECMO catheter should be checked at least once a week (F) | | | | | | | | | 76 | | 50.7 | | |
| 39 | In order to measure the oxygen saturation of the patient, care should be taken to always measure from the same finger (F) | | | | | | | | | 124 | | 82.7 | | |
| 40 | Face masks and nasal cannulas are applied alternately to prevent the formation of pressure sores that may occur on the face in newborns receiving oxygen therapy. (T) | | | | | | | | | 125 | | 83.3 | | |
| 41 | I do not alternate between face masks and nasal cannulas to prevent nasal and facial pressure sores in children and adults receiving oxygen therapy (F) | | | | | | | | | 104 | | 69.3 | | |
| 42 | If physical restraints have been applied to patients, the condition of the skin should be evaluated every 2 hours (T) | | | | | | | | | 125 | | 83.3 | | |
| 43 | There is no need to check the skin condition of a patient with an endotracheal tube (F) | | | | | | | | | 141 | | 94.0 | | |
| 44 | Protective dressing should be applied to prevent pressure injury associated with the medical device. (T) | | | | | | | | | 133 | | 88.7 | | |
| 45 | The drain of the patient with surgical drainage should be removed as soon as possible without the need for a doctor's request (F) | | | | | | | | | 131 | | 87.3 | | |
| **Statements Regarding Attitude** | | **Absolutely I agree** | | **I agree** | | **I'm undecided** | | **I do not agree** | | | **I strongly disagree** | | |  |
|  | | **n** | **%** | **n** | **%** | **n** | **%** | **n** | **%** | | **n** | | **%** |  |
| 1- Preventing MDRPIs are difficult | | 27 | 18.0 | 90 | 60.0 | 10 | 6.1 | 20 | 13.3 | | 3 | | 2.0 |  |
| 2- It is the duty of the doctor to prevent MDRPIs | | 27 | 18.0 | 67 | 44.7 | 45 | 30.0 | 11 | 7.3 | | - | | - |  |
| 3- MDRPIs are less important than other pressure injuries | | 27 | 18.0 | 72 | 48.0 | 43 | 28.7 | 8 | 5.3 | | - | | - |  |
| 4- No clinical protocols are needed to prevent MDRPIs | | 19 | 12.7 | 92 | 61.3 | 22 | 14.7 | 17 | 11.3 | | - | | - |  |
| 5-Preventing MDRPIs are not a priority for nurses | | 27 | 18.0 | 81 | 54.0 | 30 | 20.0 | 12 | 8.0 | | - | | - |  |
| 6- MDRPIs heal spontaneously | | 37 | 24.7 | 97 | 64.7 | 10 | 6.7 | 6 | 4.0 | | - | | - |  |
| 7-The occurrence of MDRPIs in the patient is an indicator of nursing quality. | | 1 | 0.7 | 114 | 76.5 | 19 | 12.7 | 6 | 4.0 | | 9 | | 6.0 |  |

**Abbreviations:** T = true statement; F = false statement. For each knowledge item, the table reports the number and percentage of participants who gave the correct response
