# Supplementary material for: Nurses' knowledge and attitudes towards medical device-related pressure injuries in intensive care and surgical units: Cross-sectional study
Source: PLoS One. 2026 Aug 19;21(8):e0356550. doi: 10.1371/journal.pone.0356550 (PMC13489443; doi:10.1371/journal.pone.0356550)
Supplement: Supplementary file 2 [file pone.0356550.s003.DOCX]

S3 Codebook. Variable definitions and scoring information

This file provides the variable definitions and coding information for the de-identified dataset submitted as Supporting Information for the manuscript entitled “Nurses' Knowledge and Attitudes Towards Medical Device-Related Pressure Injuries in Intensive Care and Surgical Units: A Cross-Sectional Study.”

Each row represents one participant (n = 150).

The dataset includes demographic and professional variables, questionnaire item responses, and total scores used in the reported analyses. The variables are defined as follows:

- agegrup: age category (1 = 18–29 years, 2 = 30–39 years, 3 = 40–49 years)
- gender: participant gender (1 = Male, 2 = Female)
- education: education level (1 = High school, 2 = Bachelor’s degree, 3 = Postgraduate degree)
- department: working department (1 = Adult ICU, 2 = Newborn ICU, 3 = Child ICU, 4 = Surgical services)
- workexperience: years of professional work experience in nursing
- receviededucation: previous MDRPI training (1 = No, 2 = Yes)

Knowledge questionnaire variables:
- diagnosis1–diagnosis4: item responses for the “description/diagnosis” knowledge subdimension
- risk1–risk21: item responses for the “risk factors” knowledge subdimension
- prevention1–prevention20: item responses for the “prevention and treatment” knowledge subdimension

Attitude questionnaire variables:
- Attitude1–Attitude7: item responses for the attitude section of the MDRPIKAQ

Total score variables:
- diagnosistotal: total score for the diagnosis/description knowledge subdimension
- risktotal: total score for the risk factors knowledge subdimension
- preventiontotal: total score for the prevention and treatment knowledge subdimension
- knowledgetotal: total knowledge score
- attiduetotal: total attitude score

Scoring:
- Knowledge item variables are coded according to the final scoring used in the submitted analysis dataset.
- knowledgetotal was calculated from diagnosis1–diagnosis4, risk1–risk21, and prevention1–prevention20. Higher scores indicate greater knowledge regarding MDRPI prevention.
- attiduetotal was calculated from Attitude1–Attitude7 according to the final scoring procedure used in the manuscript. If reverse coding was applied to any attitude items, this is reflected in the final shared dataset.

Age was retained in grouped form in the shared de-identified dataset (18–29, 30–39, and 40–49 years). Analyses involving age in the manuscript were based on these categories.

The dataset is de-identified and contains no direct personal identifiers.
