# Supplementary material for: Nurses' knowledge and attitudes towards medical device-related pressure injuries in intensive care and surgical units: Cross-sectional study
Source: PLoS One. 2026 Aug 19;21(8):e0356550. doi: 10.1371/journal.pone.0356550 (PMC13489443; doi:10.1371/journal.pone.0356550)
Supplement: Supplementary file 3 [file pone.0356550.s004.DOCX]

| **Item No** | **Recommendation** | **Reported in Manuscript (Page/Section)** |
| --- | --- | --- |
| **1a** | Indicate the study design with a commonly used term in the title or the abstract | Title page; Abstract (“descriptive cross-sectional study”) |
| **1b** | Provide an informative and balanced summary of what was done and what was found | Abstract |
| **2** | Explain the scientific background and rationale for the investigation being reported | Introduction |
| **3** | State specific objectives, including any prespecified hypotheses | End of Introduction (last paragraph: study aim) |
| **4** | Present key elements of study design early in the paper | Methods – *Study design and setting* |
| **5** | Describe the setting, locations and relevant dates, including periods of recruitment and data collection | Methods – *Study design and setting* (state hospital; units; 15 Nov–30 Dec 2022) |
| **6a** | Give the eligibility criteria, and the sources and methods of selection of participants | Methods – *Participants* (Inclusion and exclusion criteria) |
| **6b** | For matched studies, give matching criteria and number of exposed/unexposed | Not applicable (cross-sectional study; no matching design) |
| **7** | Clearly define all outcomes, exposures, predictors, potential confounders, and effect modifiers | Methods – *Data collection tools* (Nurse Identification Form; MDRPIKAQ details) |
| **8** | For each variable of interest, give sources of data and details of methods of assessment (measurement) | Methods – *Data collection tools* (description of MDRPIKAQ structure, scoring, validity, reliability) |
| **9** | Describe any efforts to address potential sources of bias | Discussion – *Limitations* paragraph (single-center, self-report, cross-sectional, possible response bias) |
| **10** | Explain how the study size was arrived at | Methods –All eligible nurses were invited (N=420), and 150 participated; the final sample exceeded the minimum required sample size (n=125) calculated by G*Power) |
| **11** | Explain how quantitative variables were handled in the analyses. If applicable, describe which groupings were chosen and why | Methods – *Statistical analysis* (description of tests; handling of total and subscale scores) |
| **12a** | Describe all statistical methods, including those used to control for confounding | Methods – *Statistical analysis* (ANOVA, t tests, correlations, linear regression) |
| **12b** | Describe any methods used to examine subgroups and interactions | Methods – *Statistical analysis* (comparisons by age, gender, education, unit, experience; regression models) |
| **12c** | Explain how missing data were addressed | Methods – *Statistical analysis* (complete-case analysis; all 150 questionnaires included) / Results – description that all 150 nurses participated |
| **12d** | If applicable, describe analytical methods taking account of sampling strategy or loss to follow-up | Not applicable (no follow-up; cross-sectional survey with single time-point measurement) |
| **12e** | Describe any sensitivity analyses | Not applicable (no sensitivity analyses performed) |
| **13a** | Report numbers of individuals at each stage of study (for example, numbers potentially eligible, examined for eligibility, included in the study) | Methods – *Study population and sample*; Fig 1 – STROBE flow chart of sample selection |
| **13b** | Give reasons for non-participation at each stage | Methods – *Non-participation was recorded as refusal or being unavailable/not eligible during the data collection period; detailed reasons were not systematically collected* |
| **14a** | Give characteristics of study participants (e.g. demographic, clinical, social) and information on exposures and potential confounders | Results – *An examination of nurses’ demographic and professional characteristics*; Table 1 |
| **14b** | Indicate number of participants with missing data for each variable of interest | Results – Text and tables (all 150 included; no missing data reported for main variables) |
| **15** | Report numbers of outcome events or summary measures | Results – Table 2 (knowledge and attitude scores); Supporting Information S1 Table (item-level correct response frequencies) |
| **16a** | Give unadjusted estimates and, if applicable, confounder-adjusted estimates and their precision (e.g. 95% confidence interval). Make clear which confounders were adjusted for and why they were included | Results – Table 4 (comparisons by demographic/professional characteristics); Results – *Predictors of MDRPIKAQ knowledge and attitude scores*; Fig 2 (linear regression, standardized beta coefficients and p-values; confidence intervals reported in text) |
| **16b** | Report category boundaries when continuous variables were categorized | Methods – *Study population and sample* and *Data collection tools* (age groups, years of experience, education levels, unit types as categories) |
| **16c** | If relevant, consider translating estimates of relative risk into absolute risk for a meaningful time period | Not applicable (cross-sectional survey; no risk/time estimates) |
| **17** | Report other analyses done (e.g. analyses of subgroups and interactions, and sensitivity analyses) | Results – Regression analysis and subgroup comparisons (age, gender, education, unit, training) |
| **18** | Summarise key results with reference to study objectives | Discussion – Opening paragraphs; Conclusion section (summary of knowledge and attitude levels and predictors) |
| **19** | Discuss limitations of the study, taking into account sources of potential bias or imprecision. Discuss both direction and magnitude of any potential bias | Discussion – *Limitations of the Study* (single center, limited generalizability, cross-sectional design, self-report data) |
| **20** | Give a cautious overall interpretation of results considering objectives, limitations, multiplicity of analyses, results from similar studies, and other relevant evidence | Discussion – Final paragraphs before Conclusion (comparison with previous studies; contextual explanations; interpretation in light of limitations) |
| **21** | Discuss the generalisability (external validity) of the study results | Discussion – *Limitations and generalizability* (organizational culture, staffing patterns, educational practices, single-hospital setting) |
| **22** | Give the source of funding and the role of the funders for the present study and, if applicable, for the original study on which the present article is based | End of manuscript – Funding / Acknowledgments section (no external funding; funders had no role) |
